# Enhancement of Black Tea Aroma by Adding the *β*-Glucosidase Enzyme during Fermentation on Black Tea Processing

**DOI:** 10.1155/2021/5542109

**Published:** 2021-08-09

**Authors:** Supriyadi Supriyadi, Alfrista Ruri Nareswari, Aprilia Fitriani, Rachmad Gunadi

**Affiliations:** ^1^Department of Food and Agriculture Products Technology, Faculty of Agricultural Technology, Universitas Gadjah Mada, Yogyakarta 55281, Indonesia; ^2^Food Technology, Faculty of Life Science, Surya University, M.H. Thamrin Street KM 2.7 Grand Serpong Mall 1st Floor F8 and F9 Units, North Panunggangan, Pinang, Tangerang City, Banten 15143, Indonesia; ^3^Department of Soil Science, Faculty of Agriculture, Gadjah Mada University, Yogyakarta, Indonesia

## Abstract

Black tea aroma is one of the essential attributes in determining the quality of black tea. *β*-Glucosidases were investigated for their ability to enhance the aroma of black tea by hydrolyzing the glycoside compound. The addition of *β*-glucosidase was done by dissolving the enzyme on a sodium citrate buffer (pH 5.0), which was then sprayed on tea leaves during black tea processing. The *β*-glucosidase treatment significantly increases the volatile compound from glycoside precursors such as linalool, geraniol, and methyl salicylate. Moreover, the volatile compound from carotenoid and lipid precursors (nerolidol and *β*-cyclocitral) was also increased with *β*-glucosidase treatment.

## 1. Introduction

Tea aroma is one of the essential properties which are decisive for the quality of the final product. The tea product aroma can be formed through various precursors such as carotenoids, fatty acids, terpenoids, phenylpropanoid/benzenoid, glycoside hydrolysis, and the Maillard reaction [1]. In black tea, aroma formation occurs due to oxidation and degradation of fatty acids and hydrolysis of glycosides [2]. *cis*-Jasmone and methyl jasmonate, which are derived from fatty acids, have fresh floral odors. But most of the other compounds derived from fatty acids are unpleasant, such as (Z)-2-hexenal and (Z)-3-hexenal, which have a grassy odor, and (E,E)-2,4-heptadienal and 2-butanone with a fatty aroma [3]. Apart from the oxidation and degradation of fatty acids, the formation of aroma in black tea also occurs due to glycoside hydrolysis. The injured tea leaf tissue will release enzymes into the cell walls to hydrolyze the glycosidic bond and release volatile compounds [4]. Most of the volatile compounds formed by glycoside hydrolysis have a pleasant aroma, such as methyl salicylate (fresh, sweet), *β*-damascenone (sweet, rose-like), and 2,5-dimethyl-4-hydroxy-3(2H)-furanone (caramel-like, sweet).

*β*-Glucosidase is one of the glycosidase enzymes that can hydrolyze glycosidic bonds [5]. The *β*-glucosidase enzyme could hydrolyze glycosides and release various free volatile compounds such as linalool and geraniol [6]. These compounds have a fruity floral odor and contribute significantly to black tea [7]. However, the endogenous enzyme *β*-glucosidase has not optimally hydrolyzed all of the glycosides present in tea leaves. Glycosides such as benzyl *β*-D-glucopyranoside, 2-phenylethyl *β*-D-glucopyranoside, (Z)-3-hexenyl-*β*-D-glucopyranoside, geranyl *β*-D-glucopyranoside, and linalyl *β*-D-glucopyranoside are still detectable in black tea products. Thus, the addition of the *β*-glucosidase enzyme in the black tea processing needs to be done to maximize the hydrolysis of glycosides in tea leaves to produce black tea products with a better aroma.

The enzyme *β*-glucosidase is widely used in fermented beverage products such as wine, oolong tea, and black tea. The *β*-glucosidase enzyme in black tea infusion increased the concentration of linalool and geraniol compounds by 10% and 18.5%, respectively [8]. The concentration of methyl salicylate and Z-3-hexenol in steeping black tea increased by 2.14% and 8.24%, respectively, with the addition of the *β*-glucosidase enzyme [9]. However, it is unclear how *β*-glucosidase could improve the aroma of black tea during processing. Therefore, the effect of adding the *β*-glucosidase enzyme, especially on aroma and other chemical components, during the black tea processing needs to be studied further.

## 2. Materials and Methods

### 2.1. Materials

Fresh tea leaves (PGL 15 clones) with shoot tips and two leaves underneath (p+2) were plucked in August 2020 from block Sanderan (1000 above sea level) in the Pagilaran plantation site, Batang, Central Java. The *β*-glucosidase enzyme (400 U/gr) was from Xi'an Geekee Biotech (China); *p*-nitrophenyl-*β*-D-glucopyranoside (*p*-NPG) and *p*-nitrophenol (*p*NP) were from Shanghai Kayi Chemical (China); the other materials used are as follows: acetone (Merck), polyvinylpolypyrrolidone (PVPP) (Merck), sodium carbonate, Folin-Ciocalteu reagent, catechol (Merck), oxalic acid, sodium hydrogen carbonate, and ethyl acetate.

### 2.2. Methods

#### 2.2.1. Preparation of Acetone Powder

The preparation of acetone powder is based on a previous study [10]. Around 20 gr fresh tea leaves were homogenized with cold acetone (400 mL; -20°C), and the suspension was filtered on a vacuum filter (Whatman filter paper no. 44) and then washed repeatedly three more times with cold acetone (200 mL) until colorless. Finally, the resultant acetone powder was dried in a desiccator. The dried powder was stored at -20°C.

#### 2.2.2. Preparation of Crude Enzyme Solution

The crude enzyme solution was prepared with 0.8 gr acetone powder, 0.4 gr PVPP, and 20 mL of a 50 mM sodium citrate buffer (pH 5.0). The mixture was centrifuged at 10000g for 25 min at 4°C. The supernatant was used as the crude enzyme solution for the assay of *β*-glucosidase and polyphenol oxidase activity [10].

#### 2.2.3. Assay of *β*-Glucosidase Activity

A substrate (*p*-NP-*β*-D-glucopyranoside) was prepared in a 50 mM sodium citrate buffer (pH 5.0). A 200 *μ*L substrate was incubated at 37°C for 5 min; then, 100 *μ*L of the enzyme was added, and the reaction mixture was incubated at 37°C for 15 min. The reaction was stopped by adding 1.4 mL of sodium carbonate solution (0.2 M). The resulting yellow color was measured at 420 nm by a spectrophotometer using *p*-nitrophenol as the standard. One unit of *β*-glucosidase activity is defined as the amount of enzymes that hydrolyzed 1 *μ*mol of the substrate/min at 37°C [10].

#### 2.2.4. Preparation of Black Tea

Black tea was made manually; fresh tea leaves (PGL 15 clones) that have been plucked were immediately subjected to the withering process using a withering machine in the factory. The process took about 12-15 hours. The following method was rolling and crushing tea shoots manually; after obtaining rolled and smaller tea leaf shoots, they are left for 2 hours at room temperature with periodic reversals. This process is also called enzymatic oxidation. The *β*-glucosidase enzyme (25 mg/10 mL) was added at the beginning of the enzymatic oxidation process. After 2 hours, the enzymatic oxidation process (fermentation) was immediately stopped by pan frying or roasting at 100 ± 5°C for 10 minutes. The final process was sieving the dry black tea to obtain the uniform size.

#### 2.2.5. Identification of Volatile Compounds in Black Tea

Based on the previous study, identification of aroma compounds in black tea was carried out [11] with modifications. Briefly, 2 gr of the ground dried black tea sample and 0.5 *μ*L of ethyl decanoate (as the internal standard (IS)) were mixed in a 22 mL vial. Subsequently, the vial was placed in an oven at 80°C for 15 min to balance the headspace gas. Afterward, a 50/30 *μ*m divinylbenzene/carboxen/polydimethylsiloxane (DVB/CAR/PDMS) 2 cm fiber was inserted into the headspace vial, and the volatiles were absorbed for 30 min under an 80°C oven condition. Volatile compounds of black tea samples were analyzed using the GC Agilent 7890A instrument with an MS Agilent 5975 XL EI/CI detector. Helium gas is carrier gas with a flow rate of 0.8 mL/min. Desorption of volatiles from samples was accomplished by inserting the SPME needle for 10 minutes at the injection temperature of 250°C, and the splitless method was used in this analysis. The oven temperature was set in a gradient with the initial temperature setting of 50°C held for 3 minutes, then increased to 190°C at a rate of 5°C/min, then improved to 220°C at a rate of 4°C/min, and held for 3 min. The mass scan range was set to *m*/*z* 29-550. The GC-MS instrument was coupled with a mass spectrometer detector with an HP-5 capillary column (Agilent, USA; 30 m, inner diameter 0.25 mm, and layer thickness 0.25 *μ*m). Chromatograms were evaluated with Xcalibur software version 1.4 (Thermo Fisher Scientific), and mass spectra were matched with NIST Library version 2005. All experiments were repeated three times. Component identification used mass spectral references in the NIST Library 2005 and retention indices (RI) [12]. RI was calculated using standard alkanes (C_9_-C_20_) under the same conditions as the sample analysis conditions.

## 3. Result and Discussion

### 3.1. The Activity of the *β*-Glucosidase Enzyme during Black Tea Processing

**Figure 1:**
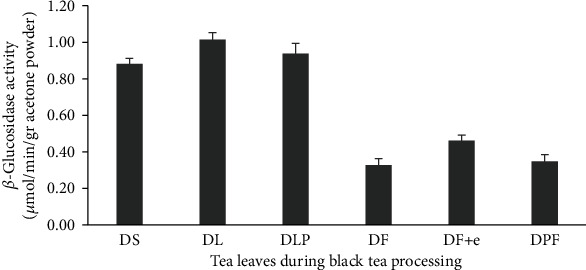
Changes of *β*-glucosidase during the black tea processing. DS: fresh leaves; DL: withered leaves; DLP: factory withered leaves; DF: fermented leaves; DF+e: fermented leaves with the *β*-glucosidase enzyme; DPF: fermented factory leaves. The error bars represent the standard deviation of measurements for five samples.

Most enzymes are proteins that can accelerate or catalyze a chemical reaction and produce a product. The activity of an enzyme is closely related to the amount of products made in units of time. The activity of the *β*-glucosidase enzyme during the black tea processing can be seen in Figure 1.

Figure 1 shows that the *β*-glucosidase enzyme activity in PGL 15 fresh leaves (P15) was 0.80 ± 0.03 *μ*mol/min/gr acetone powder and increased significantly at the withering stage (P15 L) to 0.93 ± 0.05 *μ*mol/min/gr acetone powder. Compared to the P15 L, *β*-glucosidase enzyme activity in factory withered leaves (DLP) was not significantly different, namely, 0.91 ± 0.05 *μ*mol/min/gr acetone powder. The same condition also occurred in the previous study [10], where the *β*-glucosidase enzyme activity was highest during withering. In this study, the withering stage was carried out for 15 hours. The increased concentration of cell fluids during the withering process also increased enzyme concentration, resulting in improved enzyme activity [13]. At the fermentation stage, the activity of the *β*-glucosidase enzyme in the sample of PGL 15 (P15 F) and PGL 15 leaves with *β*-glucosidase enzyme treatment (P15+e F) decreased significantly to 0.33 ± 0.04 *μ*mol/min/gr acetone powder and 0.46 ± 0.03 *μ*mol/min/gr acetone powder. The same thing also happened in the fermentation of the leaves from the factory (DPF), which was 0.35 ± 0.04 *μ*mol/min/gr acetone powder. This decrease in enzyme activity is probably due to the interaction between the enzyme and the substrate. So, the amount of substrates decreases and results in reduced activity of the *β*-glucosidase enzyme. This result is supported by the number of substrates (glycoside compounds) significantly reduced at the fermentation stage [14, 15].

At the fermentation stage, PGL 15 clones treated with the *β*-glucosidase enzyme (P15+e F) had a higher amount of enzyme activity compared to PGL 15 clones without enzyme treatment (P15 F). This phenomenon can occur because the enzyme is still able to react with the remaining substrates. The endogenous enzymes in tea leaves could not hydrolyze the substrate completely during the tea processing [16]. So more substrates will be hydrolyzed by adding the *β*-glucosidase enzyme.

### 3.2. Effect of the *β*-Glucosidase Enzyme on Glycosidic Bound Volatile Compounds

The addition of the *β*-glucosidase enzyme is often used to increase the aroma in fermented beverages such as wine and black tea. The effect of adding the *β*-glucosidase enzyme in making black tea can be seen in Table 1.

As many as 13 volatile compounds from the hydrolysis of glycosides were identified in the black tea sample. As a comparison, volatile compounds in black tea from the processing factory of PT Pagilaran were identified. Several volatile compounds were detected. 3-Hexenol is one of the volatile compounds derived from the hydrolysis of the glycosidic bonds in glycosides. This compound is a joint compound found in tea and has a fresh green odor. The volatile 3-hexenol compound is released from glycoside (Z)-3-hexenyl-*β*-D-glucopyranoside, hydrolyzed by the *β*-glucosidase enzyme, leaving *β*-D-glucopyranoside as residual sugar [6, 17]. In the THE sample, as much as 11.60 ppb was detected, twice as much as that of the THK sample detected at 5.31 ppb. The result shows that the addition of the *β*-glucosidase enzyme can hydrolyze more glycosides. In comparison, the factory black tea was seen to be as much as 19.70 ppb. As discussed by Ni et al., 3-hexenol can be isomerized to 2-hexenol [17], but this compound was not detected in the black tea sample in this study.

Other alcohol compounds detected in the black tea sample were benzyl alcohol and phenylethyl alcohol. Ho et al. explained that phenylethyl alcohol and benzyl alcohol are volatile compounds released due to the hydrolysis of the glycosidic bonds [4]. Phenylethyl alcohol is produced from the hydrolysis of phenylethyl-*β*-D-glucopyranoside and has a floral rose odor. Phenylethyl alcohol has a higher concentration in black tea with the addition of *β*-glucosidase enzyme (THE), namely, 18.40 ppb, compared to without the enzyme (THK), which is 12.97 ppb.

Benzyl alcohol is also a volatile compound from glycoside precursors. Benzyl alcohol is released from hydrolysis of the benzyl *β*-D-glucoside compound by the *β*-glucosidase enzyme and has a floral rose odor [6]. However, this study shows that the benzyl alcohol in black tea with the addition of the *β*-glucosidase enzyme (THE) has a lower concentration when compared to THK. This result may occur because the addition of the *β*-glucosidase enzyme can accelerate the enzymatic oxidation reaction so that many benzyl alcohol compounds have been converted into benzaldehyde [18].

Hydrolysis of glycosides released volatile monoterpene alcohol compounds such as geraniol (sweet, honey-like), linalool (floral), and linalool oxide (sweet, floral, creamy) [4, 19, 20]. In this study, the monoterpene alcohol compounds detected were linalool, linalool oxide, *trans*-linalool oxide, *cis*-linalool oxide, epoxylinalool, geraniol, and *cis*-geraniol. These compounds have higher concentrations in black tea with the addition of the *β*-glucosidase enzyme (THE) compared to black tea without the addition of the *β*-glucosidase enzyme (THK). This finding proves that the addition of the *β*-glucosidase enzyme in the early stages of fermentation can increase the aroma of black tea. Linalool and geraniol compounds significantly contribute to the aroma of tea products because linalool and geraniol, apart from having a pleasant odor, have a low threshold of 6 ppb and 7 ppb in water [7].

Besides volatile alcohol compounds, the breakdown of glycosidic bonds also produces nonalcoholic volatile compounds. *β*-Damascenone is one of the nonalcoholic compounds identified in the black tea sample in this study. This compound is hydrolyzed from 3-hydroxy-*β*-damascone. This present study reports that *β*-damascenone had a slightly lower concentration in the sample with the addition of the *β*-glucosidase enzyme (THE) than the sample without the addition of the *β*-glucosidase enzyme (THK). As discussed by Kinoshita et al., the release of *β*-damascenone was influenced by pH. The lower the pH, the more optimal the hydrolysis will be [21]. From this statement, the addition of the *β*-glucosidase enzyme can increase the pH of the fermented leaves so that the hydrolysis process is slightly inhibited.

Another volatile compound derived from the hydrolysis of glycosides is benzaldehyde. Although benzaldehyde has the pleasant odors of sweet, almond, and cherry notes, benzaldehyde does not contribute too much to the aroma of tea because of its high threshold of 750.9 ppb [22]. However, benzaldehyde is the main benzenoid in tea. There are two possibilities for benzaldehyde formation. The first is through the oxidation of benzyl alcohol [18], and the second is the hydrolysis of prunasin glycosides [12, 23]. The addition of the *β*-glucosidase enzyme can increase the hydrolysis process of prunasin so that it releases free benzaldehyde compounds. This is evidenced by the higher concentration of benzaldehyde in samples with the addition of the *β*-glucosidase enzyme (THE) than in samples without the addition of the *β*-glucosidase enzyme (THK).

Another nonalcoholic compound identified in this study is methyl salicylate. Methyl salicylate is produced by hydrolysis of the glycoside compound MeSA 2-O-*β*-D-xylopyranosyl-(1-6)-*β*-D-glucopyranoside (gaultherin) [24]. Su et al. [9] explained that the addition of the *β*-glucosidase enzyme could increase the concentration of methyl salicylate by 2%. But in this study, the concentration of methyl salicylate increased significantly in black tea samples with *β*-glucosidase enzyme treatment up to 35.19 ppb, while in black tea without *β*-glucosidase enzyme treatment, it was only 5.6 ppb. Based on the research of Zhu et al., methyl salicylate has a threshold of 40 ppb [25]. The addition of the *β*-glucosidase enzyme in this study resulted in a methyl salicylate concentration that was almost close to the threshold of the compound. This further strengthens the fact that premium black tea can be produced by adding the *β*-glucosidase enzyme to black tea processing.

### 3.3. The Volatile Compounds Identified in Black Tea

Apart from the hydrolysis of glycosides, the addition of the *β*-glucosidase enzyme also increases the concentration of volatile compounds from other precursors. Overall, the volatile compounds detected in black tea can be seen in Table 2.

A total of 131 volatile compounds were identified in black tea consisting of 17 alcohol compounds, ten ketones, seven ionone derivatives, eight esters, 16 aldehydes, 54 hydrocarbons, six phenolic compounds, two furanoids, four nitrogen compounds, two acid compounds, one sulfur compound, and four other compounds. Overall, the volatile compounds formed are products of enzymatic degradation of fatty acids, carotenoids, hydrolysis of glycosidic bonds, and Maillard reaction results.

Several compounds also increased from the volatile compounds detected in black tea due to the addition of *β*-glucosidase enzyme treatment. In black tea, with the addition of the *β*-glucosidase enzyme, 2-methylbutanol with a roasted wine and fruity odor increased significantly. The concentration of 2-ethylhexanol, which has a fresh, floral, sweet citrus odor, was also increased in black tea with the enzyme *β*-glucosidase. The alcohol group compound, which also increases due to the addition of the *β*-glucosidase enzyme, is nerolidol. Nerolidol is a volatile compound from carotenoid precursors with a floral green and citrus odor. In oolong tea, nerolidol is formed due to the photooxidation of phytofluene [26]. But in this study, the nerolidol compound also increased in black tea with the addition of *β*-glucosidase enzyme treatment.

Many aldehyde compounds are found in the black tea aroma and have a significant contribution. The primary formation of black tea aroma is from glycoside hydrolysis and lipid degradation [2]. Aldehyde compounds from lipid precursors that increase with the treatment of the *β*-glucoside enzyme include 2-methylpropanal with a green floral odor, 2-methylbutanal with a musty, cocoa, coffee, and nutty odor, and nonanal with a rose, fresh orris, and orange peel odor. Not all volatile compounds from lipid precursors have pleasant scents. Some of them have fatty aromas such as hexanal (fresh green, grassy), E-2-hexenal (green, banana, fatty, and cheesy), and (E,E)-2,4-heptadienal (fatty, green, and oily). The aldehyde class of compounds is also divided into aromatic aldehydes and terpenoid aldehydes. Aromatic aldehyde compounds such as benzeneacetaldehyde with a floral, sweet, clover, honey, and cocoa odor in black tea with *β*-glucosidase enzyme (THE) treatment had the highest concentration in the three black tea samples, namely, 37.18 ppb. Likewise, terpenoid aldehyde compounds include *β*-cyclocitral (sweet, fruity, tropical, saffron, and rose odor) with a concentration of 5.22 ppb. Zhang et al.'s study also showed an increase in the concentration of aromatic aldehyde compounds due to the *β*-glucosidase enzyme treatment [27].

Terpenoid hydrocarbon compounds increased with the addition of the *β*-glucosidase enzyme such as *o*-xylene with a geranium odor, D-limonene with a citrus, fresh, and sweet aroma, *α*-farnesene with a citrus, lavender, bergamot, and green scent, and *γ*-cadinene with a woody and herbal odor. These compounds had a higher concentration in the THE sample than in the THK sample. *α*-Farnesene and geranyl acetone from the ketone group are formed due to the photooxidation of phytofluene [26].

The furanoid compounds detected in black tea are furan, 2-pentyl- and furan, 3-phenyl-. Furan compound 2-pentyl- with a fruity odor has the highest concentration in the THE sample, namely, 5.06 ppb. Meanwhile, furan, 3-phenyl- with a floral odor was not detected in THK samples but detected in THE samples with a concentration of 1.73 ppb. However, it cannot be concluded that the increase in the concentration of furan, 3-phenyl- is the result of the addition of the *β*-glucosidase enzyme. As discussed by Zheng et al. and Qi et al., some furans can be formed due to the Maillard reaction, namely, the interaction between amino acids and reducing sugars [1, 28].

## 4. Conclusion

One hundred thirty-one volatile compounds were identified in black tea, and 13 are from glycoside precursors that increased due to the addition of the *β*-glucosidase enzyme. They were 3-hexenol, benzyl alcohol, phenylethyl alcohol, linalool oxide, *trans*-linalool oxide, linalool, *cis*-linalool oxide, epoxylinalool, *cis*-geraniol, geraniol, *β*-damascenone, benzaldehyde, and methyl salicylate.

## Figures and Tables

**Table 1:** Effect of the *β*-glucosidase enzyme on glycosidic bound volatile compounds.

No.	Compound	CAS number	LRI	THK (ppb)	THE (ppb)	Odor description^∗^
1	3-Hexenol	544-12-7		5.31	11.60	Green leafy
2	Benzyl alcohol	100-51-6	1042	7.82	1.94	Floral rose
3	Phenylethyl alcohol	60-12-8	1119	12.97	18.40	Floral rose, dried rose flower
4	Linalool oxide	5989-33-3	1075	6.73	15.32	Earthy, floral sweet, woody
5	*trans*-Linalool oxide	34995-77-2	1091	8.31	41.60	Floral
6	Linalool	78-70-6	1104	4.09	13.60	Citrus floral sweet, woody
7	*cis*-Linalool oxide	5989-33-3	1174	0.82	2.21	Earthy, floral sweet, woody
8	Epoxylinalool	14049-11-7	1179	4.62	11.98	Floral honey
9	*cis*-Geraniol	106-25-2	1232	nd	1.49	Sweet, neroli citrus magnolia
10	Geraniol	106-24-1	1260	2.49	4.52	Sweet floral, fruity, citrus
11	*β*-Damascenone	23726-93-4	1386	0.86	0.80	Apple, rose, honey, sweet
12	Benzaldehyde	100-52-7	962	4.08	6.61	Sweet, almond, cherry
13	Methyl salicylate	119-36-8	1199	5.60	35.19	Wintergreen, mint

Note: THK: black tea from PGL 15 controls; THE: black tea treated with *β*-glucosidase. Ethyl decanoate was used as an internal standard. ^∗^References based on http://www.thegoodscentscompany.com/. nd: not detected.

**Table 2:** The volatile compounds identified in black tea.

No.	Compound	CAS number	LRI	THK (ppb)	THE (ppb)	Odor description^∗^
*Alcohols*						
1	2-Methylbutanol	137-32-6		6.97	21.29	Roasted wine, fruity
2	3-Hexenol	544-12-7		5.31	11.60	Green leafy
3	1-Octen-3-ol	3391-86-4	983	nd	1.35	Mushroom, earthy green
4	2-Ethylhexanol	104-76-7	1035	2.46	5.38	Citrus fresh, floral sweet
5	2-Butyloctanol	735273	1320	nd	3.05	
6	7-Methoxy-1-naphthol	67247-13-6	1377	0.49	nd	

*Aromatic alcohol*						
7	Benzyl alcohol	100-51-6	1042	7.82	1.94	Floral rose
8	Phenylethyl alcohol	60-12-8	1119	12.97	18.40	Floral rose, dried rose flower
9	2-*tert*-Butylcyclohexanol	13491-79-7	1294	1.34	1.55	Pine camphor minty, patchouli

*Terpenoid alcohol*						
10	Linalool oxide	5989-33-3	1075	6.73	15.32	Earthy, floral sweet, woody
11	*trans*-Linalool oxide	34995-77-2	1091	8.31	41.60	Floral
12	Linalool	78-70-6	1104	4.09	13.60	Citrus floral sweet, blueberry
13	*cis*-Linalool oxide	5989-33-3	1174	0.82	2.21	Earthy, floral sweet, woody
14	Epoxylinalool	14049-11-7	1179	4.62	11.98	Floral honey
15	*cis*-Geraniol	106-25-2	1232	nd	1.49	Sweet natural, neroli citrus
16	Geraniol	106-24-1	1260	2.49	4.52	Sweet floral, fruity, rose, citrus
17	Nerolidol	7212-44-4	1568	3.50	6.52	Floral green, citrus, woody

*Ketones*						
18	2,3-Octanedione	585-25-1	988	0.43	6.83	Dill, asparagus, cilantro, herbal
19	2,5,5-Trimethylcyclohex-2-enone	42747-41-1	1301	2.42	2.91	
20	2-Benzylcyclohexanone	946-33-8	1468	0.10	0.69	

*Aromatic ketone*						
21	Acetophenone	98-86-2	1062	1.76	0.78	Sweet pungent, almond, acacia
22	Camphenone, 6-	55659-42-2	1205	1.33	3.07	
23	Geranyl acetone	3796-70-1	1455	0.62	1.32	Fresh green, fruity, rose, tropical
24	2,4,5-Trimethylacetophenone	2040-07-5	1486	1.79	4.52	
25	Benzophenone	119-61-9	1634	1.12	1.05	Rose, powdery, geranium
26	4′-Benzyloxyacetophenone	782-92-3	1693	0.27	nd	
27	Hexahydrofarnesyl acetone	502-69-2	1847	nd	0.26	Jasmine, celery, woody

*Ionone derivatives*						
28	*β*-Damascenone	23726-93-4	1386	0.86	0.80	Apple, rose, honey, tobacco, sweet
29	*α*-Ionone	127-41-3	1432	0.50	0.87	Sweet, woody, floral, tropical fruity
30	Dihydro-*β*-ionone	17283-81-7	1444	nd	0.15	Earthy, woody, dry amber
31	1-(4-*tert*-Butylphenyl)propan-2-one	81561-77-5	1453	0.42	nd	
32	2,6-Di-*tert*-butylquinone	719-22-2	1471	1.59	2.56	
33	*α*-Isomethyl ionone	127-51-5	1481	0.70	0.40	Sweet orris, powdery, floral, woody
34	*trans*-*β*-Ionone	79-77-6	1490	4.02	7.04	Dry powdery, floral, woody, orris

*Esters*						
35	Methyl caprylate	111-11-5	1126	nd	3.38	Green, sweet orange
36	*cis*-3-Hexenyl-*α*-methylbutyrate	53398-85-9	1238	nd	0.62	Fresh, green apple, sweet, pear
37	Isobornyl acetate	125-12-2	1289	1.43	0.87	Herbal, woody, sweet
38	3-Hexenyl hexanoate	84434-19-5	1383	1.28	nd	
39	Ethyl decanoate	110-38-3	1397	2.50	2.50	Sweet, fruity, apple, grape
40	Methyl laurate	111-82-0	1529	nd	1.96	Creamy, coconut, mushroom
41	*cis*-3-Hexenyl benzoate	25152-85-6	1576	0.42	1.06	Fresh green, leafy, floral, orchid
42	Methyl palmitate	112-39-0	1928	0.24	1.01	Waxy, fatty orris

*Aldehydes*						
43	2-Methylpropanal	78-84-2		22.51	31.52	Floral green
44	2-Methylbutanal	96-17-3		17.70	44.94	Musty, cocoa, coffee, nutty
45	Hexanal	66-25-1		3.54	4.85	Fresh green, grass leafy, sweaty
46	2-Hexenal, (E)-	6728-26-3		2.15	4.63	Green banana, fatty, cheesy
47	Heptanal	111-71-7	902	5.75	4.19	Fresh green, herbal, wine-lee ozone
48	2,4-Heptadienal, (E,E)-	4313-03-05	1012	nd	1.25	Fatty green oily, cake cinnamon
49	Nonanal	124-19-6	1106	20.61	39.88	Rose, fresh orris, orange peel
50	Decanal	112-31-2	1207	0.82	0.74	Sweet, orange peel, citrus floral

*Aromatic aldehyde*						
51	Benzaldehyde	100-52-7	962	4.08	6.61	Sweet, almond, cherry
52	Benzeneacetaldehyde	122-78-1	1046	16.00	37.18	Floral, sweet, clover, honey, cocoa
53	2-Butenal, 2-phenyl	4411-89-6	1276	0.38	0.50	Beany, honey cocoa, nutty radish
54	4-Indanecarbaldehyde	51932-70-8	1306	1.09	1.68	
55	2-Hexylcinnamaldehyde	101-86-0	1753	0.33	nd	Fresh, floral green, jasmine, herbal

*Terpenoid aldehyde*						
56	*β*-Cyclocitral	432-25-7	1223	3.22	5.22	Sweet, fruity, tropical, saffron, rose
57	(R)-(+)-Citronellal	2385-77-5	1242	nd	0.31	Fresh herbal, citrus
58	Citral	5392-40-5	1274	0.39	0.29	Sharp lemon sweet

*Hydrocarbons*						
59	4-Methyldecane	2847-72-5	1023	1.44	1.19	
60	3,6-Dimethyldecane	17312-53-7	1058	1.93	2.05	
61	4-Methylundecane	2980-69-0	1059	2.39	1.65	
62	3,8-Dimethyldecane	17312-55-9	1150	1.05	0.87	
63	3-Methylundecane	1002-43-3	1171	1.99	1.85	
64	Cyclododecane	294-62-2	1192	1.44	1.27	
65	Dodecane	112-40-3	1201	30.48	31.71	
66	2-Undecene, 5-methyl-	56851-34-4	1215	1.98	1.42	
67	2-Methyldodecane	1560-97-0	1264	4.26	2.35	
68	5-Ethyl-2-methyloctane	62016-18-6	1280	4.39	2.50	
69	3,5-Dimethyldodecane	107770-99-0	1340	0.69	0.63	
70	2,3,7-Trimethyldecane	62238-13-5	1343	nd	0.24	
71	4-Ethyldecane	1636-44-8	1347	1.08	1.40	
72	3-Dodecene, (Z)-	7239-23-8	1365	nd	0.75	
73	3-Methyltridecane	6418-41-3	1371	2.48	2.39	
74	Cyclopentane, nonyl-	2882-98-6	1386	0.87	nd	
75	1-Tetradecene	1120-36-1	1392	1.82	1.66	
76	Tetradecane	629-59-4	1401	15.52	13.89	Mild waxy
77	Cyclotetradecane	295-17-0	1449	0.52	0.62	
78	4,8-Dimethyltridecane	55030-62-1	1463	1.47	1.03	
79	Pentadecane	629-62-9	1500	0.71	26.58	Waxy
80	Hexadecane	544-76-3	1541	0.69	0.87	
81	Heptadecane	629-78-7	1600	2.33	3.38	

*Aromatic hydrocarbon*						
82	Naphthalene	91-20-3	1186	12.06	12.22	Pungent, dry tarry
83	Eicosane	112-95-8	1710	0.24	0.44	Waxy
84	Cadinene	5951-61-1	1336	1.88	1.67	
85	Ionene	475-03-6	1360	nd	0.23	
86	2,6-Dimethylnaphthalene	581-42-0	1426	nd	0.54	Grass
87	1,3-Diisopropylnaphthalene	1000374-05-2	1682	0.48	nd	
88	Cadalene	483-78-3	1686	0.76	1.21	
89	1,4-Diisopropylnaphthalene	1000374-05-7	1727	0.29	0.82	
90	2,6-Diisopropylnaphthalene	24157-81-1	1730	3.60	2.26	
91	Copaene	3856-25-5	1379	1.16	1.06	Woody spicy honey
92	Modephene	68269-87-4	1383	nd	0.77	
93	Cyclosativene	22469-52-9	1374	nd	0.84	
94	Berkheyaradulene	1000373-94-1	1391	nd	1.09	
95	Caryophyllene	87-44-5	1424	2.34	2.87	Sweet, woody spice clove dry
96	Alloaromadendrene	25246-27-9	1505	0.58	0.76	Woody
97	Butylhydroxytoluene	128-37-0	1518	2.69	4.60	Mild camphor

*Terpenoid hydrocarbon*						
98	*o*-Xylene	95-47-6		7.75	9.18	Geranium
99	D-Limonene	5989-27-5	1028	0.26	0.57	Citrus orange, fresh sweet
100	*α*-Cubebene	17699-14-8	1354	0.90	0.77	Herbal waxy
101	*α*-Longifolene	475-20-7	1409	1.25	1.31	Sweet woody, rose, medical, fir
102	*ε*-Muurolene	30021-46-6	1415	1.32	2.41	
103	*β*-Cubebene	13744-15-5	1436	nd	0.26	Citrus, fruity, radish
104	*trans*-*α*-Bergamotene	13474-59-4	1438	nd	0.14	Woody, warm tea
105	*β*-Guaiene	88-84-6	1442	nd	0.27	Sweet woody, spicy, powdery
106	*β*-Bisabolene	495-61-4	1459	0.61	0.80	Balsamic, woody
107	*γ*-Gurjunene	22567-17-5	1478	nd	0.95	Musty
108	*α*-Farnesene	502-61-4	1511	1.45	3.07	Citrus, lavender, bergamot, green
109	*γ*-Cadinene	39029-41-9	1521	nd	3.26	Herbal, woody
110	*α*-Cedrene	469-61-4	1654	nd	0.36	Woody, cedar, sweet fresh
111	*α*-Longipinene	5989-0805	1658	nd	0.35	
112	Dehydro-*ar*-ionene	30364-38-6	1358	0.54	0.32	Licorice

*Phenolic compounds*						
113	*o*-Cresol	95-48-7	1165	1.85	1.15	Musty, plastic, medicinal, leathery
114	Eugenol	97-53-0	1364	1.44	0.31	Sweet spicy, clove woody
115	Methyl salicylate	119-36-8	1199	5.60	35.19	Wintergreen, mint
116	2,4-Di-*tert*-butylphenol	96-76-4	1524	2.05	2.52	
117	4-(1-Hydroxyallyl)-2-methoxyphenol	112465-50-6	1531	2.28	0.51	
118	2,4,6-Tri-*tert*-butylphenol	732-26-3	1910	nd	nd	

*Furanoid*						
119	Furan, 2-pentyl-	3777-69-3	992	2.00	5.06	Fruity, green
120	Furan, 3-phenyl-	13679-41-9	1226	nd	1.73	Floral

*Nitrogenous compounds*						
121	5-Methylthiazole	3581-89-3	1246	1.55	2.44	
122	Dihydroactinidiolide	17092-92-1	1538	0.62	nd	
123	2-Methylphenothiazine	5828-51-3	1648	nd	0.92	
124	Caffeine	58-08-2	1858	1.76	3.35	Odorless

*Acids*						
125	2-Ethylhexanoic acid	149-57-5	1139	4.47	1.46	
126	*n*-Decanoic acid	334-48-5	1283	0.56	0.44	Unpleasant, rancid, sour fatty

*Sulfur compound*						
127	2-Ethyl-5,7-dimethyl-1-benzothiophene	18428-05-2	1422	0.57	nd	

*Others*						
128	1-Oxaspiro[4.5]dec-6-ene,2,6,10,10-tetramethyl-1-oxaspiro[4.5]dec-6-ene	36431-72-8	1315	nd	0.76	Tea, herbal, green, wet tobacco leaf, woody, spicy
129	Diphenyl ether	101-84-8	1405	0.90	0.78	Geranium leaf, green
130	Cyclopropane, nonyl-	74663-85-7	1474	0.35	0.27	
131	Biphenyl-, 2,2′,5,5′-tetramethyl-	3075-84-1	1716	0.34	0.58	

Note: THK: black tea from PGL 15 controls; THE: black tea treated with *β*-glucosidase; THP: black tea from the Pagilaran factory. Ethyl decanoate was used as an internal standard. ^∗^References based on http://www.thegoodscentscompany.com/. nd: not detected.

## Data Availability

The data used to support the findings of this study are available from the corresponding author upon request.
